# Application of transthoracic echocardiography in patients receiving intermediate- or high-risk noncardiac surgery

**DOI:** 10.1371/journal.pone.0215854

**Published:** 2019-04-25

**Authors:** Hsien-Yuan Chang, Wei-Ting Chang, Yen-Wen Liu

**Affiliations:** 1 Division of Cardiology, Department of Internal Medicine, National Cheng Kung University Hospital, College of Medicine, National Cheng Kung University, Tainan, Taiwan; 2 Institute of Clinical Medicine, College of Medicine, National Cheng Kung University, Tainan, Taiwan; 3 Division of Cardiology, Department of Internal Medicine, Chi Mei Medical Center, Tainan, Taiwan; 4 Department of Biotechnology, Southern Taiwan University of Science and Technology, Tainan, Taiwan; Mayo Clinic, UNITED STATES

## Abstract

**Background:**

Cardiovascular events are the leading cause of perioperative complications among patients undergoing noncardiac surgery. However, the role of echocardiography for preoperative cardiac risk stratification prior to major noncardiac surgery is still controversial.

**Methods:**

This retrospective study included a total of 1453 patients (51% male; age, 67 ± 16) who underwent intermediate- or high-risk major abdominal surgery or orthopedic surgery at two medical centers in South Taiwan between February 2013 and June 2016. All patients underwent preoperative transthoracic echocardiography (TTE). All of the included patients were followed up for 56 days after surgery. The primary endpoints were major adverse events (MAEs), i.e., all-cause mortality and major adverse cardiovascular-cerebral events (MACCEs).

**Results:**

A total of 35 patients (2.4%) reached the primary endpoint: 24 patients (1.6%) died, and 17 patients (1.2%) had MACCEs. Patients with postsurgery MAEs had higher average E/e’ values, a lower Left ventricular (LV) ejection fraction, and higher prevalence of significant mitral regurgitation (MR) and moderate-advanced chronic kidney disease (CKD). Multivariate analysis showed that the modified Lee index and significant MR were independent prognostic predictors of MAEs.

**Conclusion:**

Preoperative identification of significant MR on TTE is associated with increased MAEs at 56 days compared with that predicted by the modified Lee index alone in patients undergoing intermediate- or high-risk noncardiac surgery.

## Introduction

Cardiovascular events are the leading cause of mortality among patients undergoing noncardiac surgery and depend on patient-related risk factors, the type of surgery, and the circumstances. Approximately 1–5% of cardiac events were reported in patients undergoing intermediate- or high-risk noncardiac surgery.[1] Transthoracic echocardiography (TTE) is a feasible and noninvasive technique used to evaluate cardiac structure and function. Nevertheless, there is no consensus regarding how to comprehensively assess perioperative risk. Several studies do not support the routine use of preoperative TTE for cardiac risk evaluation before major noncardiac surgery because echocardiographic measurements do not have better prognostic abilities than clinical risk factors.[2–5] On the other hand, some studies have demonstrated a positive correlation between a reduced Left ventricular (LV) ejection fraction (EF) and perioperative complications in patients undergoing major noncardiac surgeries.[6–10] We acknowledge that preoperative LV systolic function assessment is proven to predict perioperative outcome and long-term mortality in patients undergoing high-risk noncardiac surgery.[11] In real world practice, such as in Korea[12] or Taiwan, many surgeons and anesthesiologists have their own practical standards for requesting preoperative TTE prior to noncardiac surgery. For example, in elderly patients without a completely normal electrocardiogram or chest X-ray, preoperative TTE is commonly requested.

Moreover, although neither the American College of Cardiology and the American Heart Association (ACC/AHA) guidelines nor the European Society of Cardiology and the European Association of Anesthesiology (ESC/ESA) guidelines[1, 13] recommend routine echocardiography for assessing cardiac function and geometry before noncardiac surgery, there is no well-defined indication for preoperative TTE according to the recommendations of the American Society of Echocardiography (ASE)[14]. In short, the role of preoperative TTE for perioperative cardiac risk stratification prior to intermediate- or high-risk noncardiac surgery has not been well illustrated. As a result, we conducted this retrospective observational study to investigate the prognostic value of preoperative TTE for patients undergoing intermediate- or high-risk noncardiac surgery.

## Materials and methods

### Study design

This retrospective study included patients who underwent scheduled major noncardiac surgery (i.e., abdominal surgery and orthopedic surgery) and TTE within three months between February 2013 and June 2016 at the National Cheng Kung University Hospital and Chi-Mei Hospital in Tainan, Taiwan. This study adhered to the Declaration of Helsinki and received approval from the Human Research and Ethics Committee of National Cheng Kung University Hospital (IRB number: A-ER-105-081). Because this was a retrospective study, all the data were fully anonymized and the Human Research and Ethics Committee of National Cheng Kung University Hospital waived the requirement for informed consent (supporting information). Clinical information on comorbidities, medical history, and types of surgeries were obtained by careful review of each patient’s medical record. Based on the ESC/ESA guidelines on noncardiac surgery [1], surgical risk was estimated according to the type of surgery. Clinical risks were estimated by the modified Lee risk index, in which diabetes mellitus is used instead of treatment with insulin because the treatment of diabetes was not comprehensively recorded in our study. Therefore, the six predictors of the modified Lee index were creatinine ≥2 mg/dL, heart failure, diabetes mellitus, intrathoracic, intra-abdominal, or suprainguinal vascular surgery, history of a cerebrovascular accident or transient ischemic attack (TIA), and ischemic heart disease. The inclusion criteria were age > 18 years old, modified Lee index > 0, and moderate- to high-risk abdominal surgery or orthopedic surgery. Additionally, TTE studies were comprehensively recorded. Patients with inadequate echocardiographic image quality for analysis were excluded.

### Echocardiographic study

Prior to noncardiac surgeries, all patients were examined in the left lateral decubitus position by well-trained echocardiographers using an ultrasound system with a 3.5-MHz probe (Vivid-E9, GE Healthcare, Horten, Norway, or iE-33, Philips, Netherland). Quantifications of the LV mass index (LVMi), LVEF, and left atrial volume index (LAVi) were performed according to the ASE recommendations[15], and LVH was defined as an LVMi >115 g/m^2^ for men and >95 g/m^2^ for women. The LAVi was calculated by (A1 x A2/ L) x 8 / 3π, where L was the average LA length in the apical four- and two-chamber views. Mitral inflow measurements included early (E) and late (A) peak velocities and the E/A ratio. Pulse tissue Doppler imaging velocities were acquired at the septal and lateral mitral annuli, including the peak systolic (s’) and early diastolic (e’) velocities. The ratio of early transmitral flow to early diastolic mitral annular velocity (E/e’) was calculated from the average of the septal and lateral e’ (average E/e’ = E/[(e’_septal_+e’_lateral_)/2]). Two-dimensional grayscale images were acquired in three standard apical views: apical 2-chamber, apical 4-chamber and apical long-axis views. All images were acquired for 3 consecutive cardiac cycles and stored digitally with a frame rate of 50–90 frames/s for subsequent offline analysis. Furthermore, significant valvular heart disease was defined as at least moderate valvular heart disease. According to the 2016 ESC guidelines, an LVEF of 40–49% was defined as a mid-range EF. Therefore, we chosen an EF <40% as a variable to separate it.[16]

### Endpoints

All the included patients were followed up for 56 days after surgery. According to previous literature studying survival following noncardiac surgeries, most deaths occurred within the first 2 months[17, 18]. Therefore, we finally decided on a follow-up period of 8 weeks (56 days). The patients’ medical records were carefully reviewed throughout the follow-up period. The primary endpoint was major adverse events (MAEs), which were defined as all-cause mortality and major adverse cardiovascular-cerebral events (MACCEs). MACCEs were defined as cardiovascular death, acute coronary syndrome, hospitalization for heart failure, ventricular tachycardia, and stroke. The definitions of cardiovascular death and hospitalization for heart failure are presented in Table 1. The diagnosis of acute coronary syndrome and ventricular tachycardia was confirmed by the consulting cardiologists, and the diagnosis of stroke was confirmed by the consultant neurologists.

**Table 1:** Definitions of cardiovascular death and heart failure hospitalization.

Adverse events	Definitions
**Cardiovascular death**	**1)** death resulting from an acute myocardial infarction, or **2)** sudden cardiac death, or **3)** death due to heart failure or cardiogenic shock, or **4)** death due to stroke, or **5)** death due to other cardiovascular causes: i.e. dysrhythmia, pulmonary embolism, percutaneous transluminal cardiovascular intervention (PCI) (other than PCI related to acute myocardial infarction), aortic aneurysm rupture, or peripheral arterial disease
**Heart failure (HF) hospitalization**	An event requiring hospitalization and meeting all the following criteria: 1) An admission to an inpatient unit or a visit to an emergency department for at least a 24-hour stay 2) New or worsening clinical HF symptoms 3) Physical signs of HF 4) Need for additional or increased therapy for HF 5) Neither non-cardiac etiology nor cardiac etiology for HF signs or symptoms is identified.
**Ventricular tachycardia**	Based on electronic record of consulting cardiologists
**Acute coronary syndrome**	Based on electronic record of consulting cardiologists
**Stroke**	Based on electronic record of consulting neurologists

### Statistical analysis

All statistical analyses were performed with SPSS software version 21.0 (IBM, Armonk, NY, USA). Continuous data are presented as the mean ± standard deviation or as the median (interquartile range), depending on the distribution. Dichotomous data are presented as numbers and percentages. Comparisons were conducted using Student’s *t*-test or the Mann-Whitney U test for continuous variables with normal or nonparametric distributions, respectively. The chi-square test or Fisher’s exact test was used for categorical variables where appropriate. The Kaplan-Meier method was used with a log-rank test to compare survival rates between strata. Univariate Cox regression analysis was performed to evaluate factors associated with MAEs. Factors with *p* <0.1 in the univariate analysis were used in the multivariable Cox regression analysis to investigate risk factors for MAEs.

Because there were few events in our study, a large number of variables could not be used in the Cox regression model. Therefore, we used two models: model 1 included the modified Lee index and significant factors from echocardiography, and model 2 included the modified Lee index and significant clinical factors. A two-sided *p* <0.05 was considered statistically significant. The prognostic value of preoperative echocardiography compared with clinical risk factors was assessed in models according to variables with p <0.1 in the univariate analysis. We used the -2log likelihood ratio statistic following a χ2 distribution to evaluate the significance of improvement in condition prediction, and the p value was based on the incremental value compared to the other conditions.

## Results

**Fig 1:**
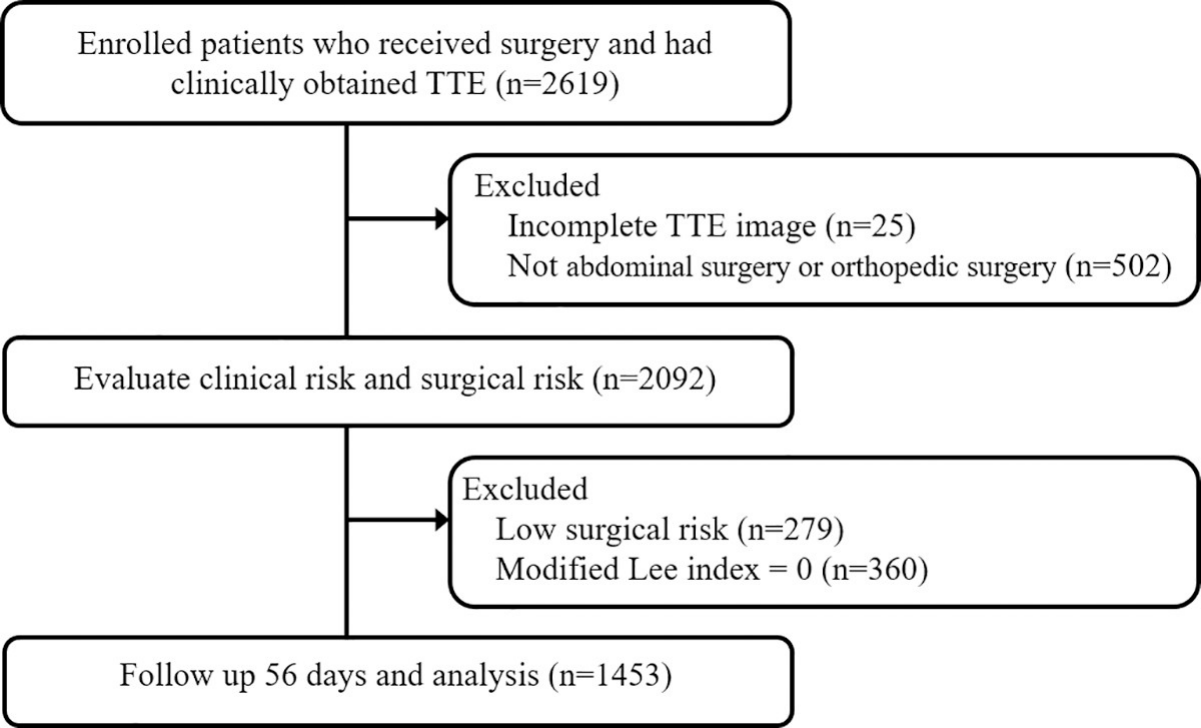
Consort diagram for patient inclusion.

This study retrospectively screened a total of 2619 patients who underwent preoperative TTE. Of these, 2092 patients underwent abdominal surgery or orthopedic surgery. A total of 279 patients with low surgical risk and 360 patients with a score of 0 on the modified Lee index were excluded. A total of 1453 patients were included in the final analysis. (Fig 1) No patient was lost to follow-up during the study period (8 weeks after major noncardiac surgery). Thirty-five patients (2.4%) had MAEs. Twenty-four patients (1.7%) died of the following causes: cardiovascular death (n = 2), infections (n = 7), liver disease (n = 1), ischemic bowel (n = 2) and other causes, such as respiratory failure, metabolic acidosis, septic shock, or unknown etiology (n = 12). Additionally, there were seventeen patients (1.2%) with MACCEs: two patients had acute coronary syndrome, six patients were hospitalized for heart failure, four patients had ventricular tachycardia, four patients had stroke, and one patient had a pulmonary embolism. Six patients had MACCEs and died. However, given the limited number of MACCEs, this outcome failed to reach statistical power. Therefore, we focused only on patients with MAEs in the following analysis. The included patients were stratified into the MAE-free group or the MAE group (Table 2). The MAE group included older patients, had a higher percentage of males, and had higher prevalence of coronary artery disease, heart failure, and moderate-advanced chronic kidney disease (CKD, defined as a creatinine clearance (CCr) < 60 ml/min). The risk of MAE was 3.3% in the high surgical risk group and 0.8% in the intermediate surgical risk group.

**Table 2:** Baseline characteristics of patients receiving intermediate or high risk non-cardiac surgery.

	MAE free	MAE	*p*
	(n = 1418)	(n = 35)	
Age (y/o)	66.8 ± 16.1	71.4 ± 12.2	0.036
Sex (Female)	699 (49.3%)	11 (31.4%)	0.037
BMI (Kg/m^2^)	24.7 ± 5.1	21.9 ± 4.1	0.001
BMI (Kg/m^2^) < 19	138 (9.7%)	7 (20%)	0.045
Diabetes mellitus	525 (37.0%)	18 (51.4%)	0.082
Hypertension	705 (49.7%)	20 (57.1%)	0.385
CAD	192 (13.5%)	11 (31.4%)	0.003
Dyslipidemia	129 (9.1%)	4 (11.4%)	0.637
HF	50 (3.5%)	4 (11.4%)	0.015
Old stroke	65 (4.6%)	1 (2.9%)	0.628
Smoking	99 (7.0%)	2 (5.7%)	0.771
Cr ≧ 2.0 (mg/dl)	130 (9.2%)	15 (42.9%)	<0.001
CCr <60 (ml/min)	591 (41.7%)	22 (62.9%)	0.012
Surgical risk			0.002
Intermediate-risk	571 (40.3%)	5 (14.3%)	
High-risk	847 (59.7%)	30 (85.7%)	
Modified Lee Index			<0.001*
1	889 (62.7%)	10 (28.6%)	
2	391 (27.6%)	12 (34.3%)	
3	113 (8.0%)	7 (20.0%)	
4	21 (1.5%)	5 (14.3%)	
5	4 (0.3%)	1 (2.9%)	

Abbreviations: BMI, body mass index; CAD, coronary artery disease; CCr, clearance of creatinine; HF, heart failure; MAE, major adverse events.

*p* value for comparison between patients in the MAE and the MAE-free groups by Student’s t-test for continuous data and chi-square test or Fisher’s exact test for categorical variables.

* *p* for trend to comparison modified Lee index between patients in the MAE and the MAE-free groups

Data are expressed as mean ± SD or number (%)

### Evaluation of cardiac function

There was no significant difference in the LV geometry, LA size and RV systolic function between the MAE-free and the MAE groups (Table 3). However, patients with postoperative MAEs had higher average E/e’ values, a lower LV ejection fraction and more significant valvular heart disease, especially mitral regurgitation (MR), than patients in the MAE-free group.

**Table 3:** Baseline echocardiography data of patients receiving intermediate or high risk non-cardiac surgery.

	MAE-free	MAE	*P*
	(n = 1418)	(n = 35)	
EDV (cm^3^)	111.1 ± 35.6	114.5 ± 52.3	0.703
ESV (cm^3^)	33.9 ± 22.6	44.5 ± 44.8	0.171
EF (%)	70.6 ± 9.4	65.3 ± 16.6	0.066
EF < 50%, n (%)	44 (3.1%)	5 (14.3%)	<0.001
EF < 40%, n (%)	18 (1.3%)	3 (8.6%)	<0.001
EF < 35%, n (%)	8 (0.6%)	3 (8.6%)	<0.001
LVMi (g/m^2^)	89.2 ± 31.0	106.4 ± 48.8	0.045
LAVi (ml)	25 ± 13	30 ± 24	0.218
E (cm/s)	72 ± 23	69 ± 24	0.503
Average e’ (cm/s)	8.1 ± 2.6	7.0 ± 1.6	<0.001
Septal e' (cm/s)	7.1 ± 2.9	6.4 ± 3.1	0.210
Lateral e' (cm/s)	9.1 ± 3.2	8.3 ± 2.6	0.161
Average E/ e'	9.5 ± 3.7	10.6 ± 4.8	0.218
Average E/ e’>14	139 (9.8%)	7 (20.0%)	0.034
Average E/ e’>15	109 (7.7%)	7 (20.0%)	0.005
RV s' (cm/s)	13.2 ± 2.8	13.6 ± 4.3	0.618
Significant VHD, n (%)	145 (10.2%)	12 (34.3%)	<0.001
Significant AS/MS	15 (1.1%)	0 (0.0%)	0.541
Significant AR/MR	102 (7.2%)	10 (28.6%)	<0.001
Significant AR	64 (4.5%)	4 (11.4%)	0.056
Significant MR	51 (3.6%)	6 (17.1%)	<0.001
Significant TR	54 (3.8%)	3 (8.6%)	0.152

Abbreviations: EDV, end-diastolic volume; ESV, end-systolic volume; EF, ejection fraction; E/e’, early trans-mitral velocity to tissue Doppler mitral annular early diastolic velocity ratio; LAVi, left atrial volume index; LVMi, left ventricular mass index; MAE, major adverse events; RV, right ventricular; s’, left ventricular systolic myocardial velocity; VHD, valvular heart disease, AS, aortic stenosis, MS, mitral stenosis, AR, aortic regurgitation, MR, mitral regurgitation, TR, tricuspid regurgitation.

p value for comparison between patients in the MAE and the MAE-free groups by Student’s t-test for continuous data and chi-square test or Fisher’s exact test for categorical variables.

Data are expressed as mean ± SD or number (%)

### Prognostic indicator stratification

**Fig 2:**
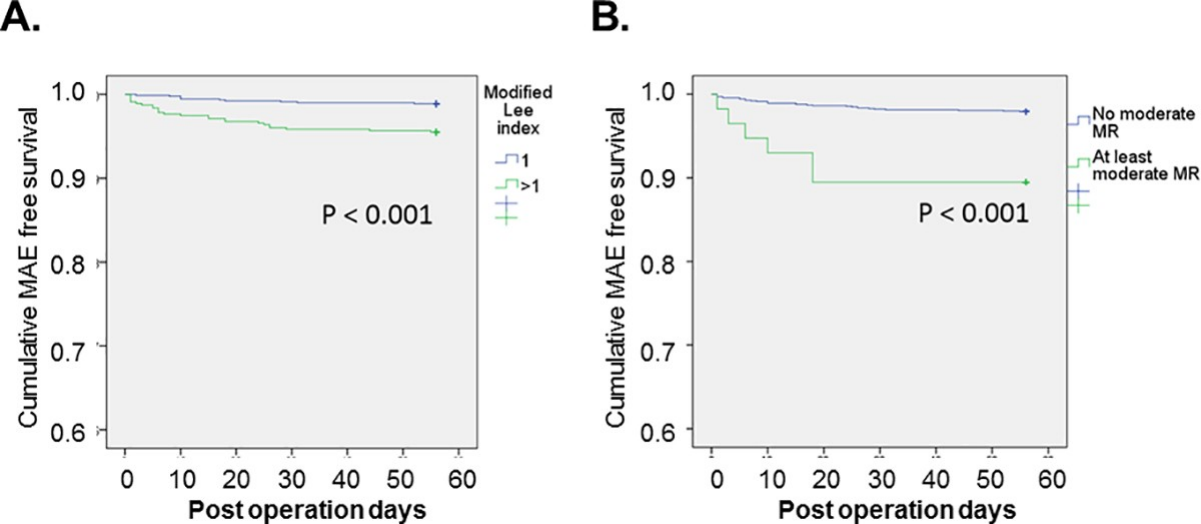
Kaplan-Meier curve of cumulative MAE-free survival. A. A modified Lee index score >1 was associated with more MAEs than a modified Lee index score = 1 (p<0.001 by log-rank test). B. Patients with at least moderate MR had more MAEs than patients without moderate MR (p < 0.001).

**Fig 3:**
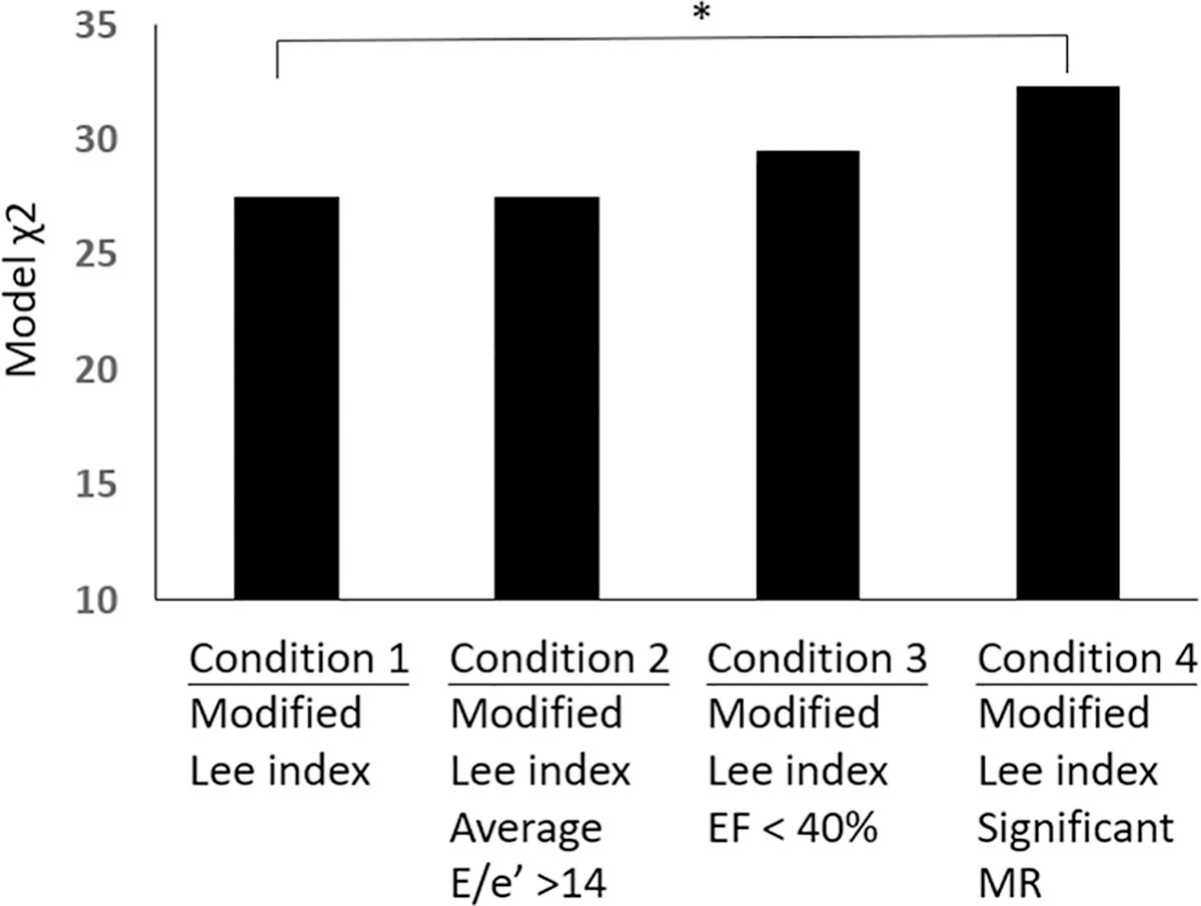
Incremental prognostic information of the modified Lee index, average E/e’ >14, EF < 40%, and significant MR. Based on a comparison of the overall log likelihood χ2 of the predictive power, there was no incremental prognostic information for MAE between Condition l and Condition 2 or between Condition 1 and Condition 3. Only Condition 4 added incremental prognostic power compared with Condition 1 (p = 0.028).

Univariate logistic regression analysis showed that the modified Lee index, age, gender, BMI< 19 kg/m2, EF < 40%, average E/e’ > 14 and significant MR were significantly associated with MAEs (Table 4). In the multivariate Cox regression analysis, we evaluate the impact of echocardiographic and clinical parameters separately. In Model 1, the modified Lee index and significant MR but not EF < 40% or average E/e’ > 14 were independent prognostic predictors of MAEs (Table 4). In Model 2, compared with age, sex, and BMI < 19 kg/m2, only the modified Lee index was significantly associated with MAEs. Furthermore, with a difference in the modified Lee index > 1 and significant MR, the Kaplan-Meier survival curves demonstrated significant differences in terms of major adverse events (Fig 2). Significant MR added incremental prognostic information to the modified Lee index for MAEs based on a comparison of the overall log likelihood χ2 of the predictive power. (Fig 3)

**Table 4:** Multivariable Cox regression model to identify factors associated with major adverse events (MAEs).

	Univariate		Multivariate			
			Model 1		Model 2	
Variables	Hazard ratio (95% CI)	*p* value	Hazard ratio (95% CI)	*p* value	Hazard ratio (95% CI)	*p* value
Modified Lee index	2.38 (1.78–3.17)	<0.001	2.22 (1.64–3.02)	<0.001	2.38 (1.78–3.18)	<0.001
Diabetes mellitus	1.79 (0.93–3.48)	0.084				
CAD	2.88 (1.41–5.89)	0.004				
HF	3.45 (1.22–9.79)	0.02				
Cr ≧ 2.0 (mg/dl)	7.00 (3.58–13.65)	<0.001				
Surgical risk	3.99 (1.55–10.28)	0.004				
EF < 40%	6.72 (2.06–21.95)	0.002	1.64 (0.39–6.83)	0.497		
Average E/ e’>14	2.42 (1.05–5.57)	0.038	1.06 (0.40–2.78)	0.913		
Significant MR	5.33 (2.21–12.84)	<0.001	3.31 (1.31–8.35)	0.011		
Age (y/o)	1.02 (0.99–1.05)	0.1			1.02 (0.99–1.04)	0.162
Sex (Female)	0.48 (0.23–0.97)	0.042			0.65 (0.31–1.37)	0.256
BMI (Kg/m^2^) < 19	2.29 (1.00–5.25)	0.05			2.26 (0.99–5.17)	0.054

Note: 1. Backward LR method. 2. Only four important factors were chosen into multivariate cox regression model because the number of MAE group was too small. DM, CAD, HF, Cr ≧ 2.0 and surgical risk were factors in modified Lee index

Abbreviations: CI, confidence interval; BMI, body mass index; CAD, coronary artery disease; DM, Diabetes mellitus; HF, heart failure EF, ejection fraction; MAE, major adverse events; MR, mitral regurgitation.

## Discussion

This study showed that significant MR identified by preoperative echocardiography may provide incremental information in patients undergoing intermediate- or high-risk major noncardiac surgeries. Our data also showed that the modified Lee index was a prognostic factor in patients undergoing intermediate-risk noncardiac surgery. According to the ESC/ESA guidelines on noncardiac surgery, patients with a Lee index score of 1, 2, and 3+ had a cardiac event risk of 0.9%, 6.6% and 11.0%, respectively[1]. According to our data, patients with a modified Lee index score of 1, 2, and 3+ had a risk of MAE of 1.1%, 2.9% and 8.6%, respectively. This association between the Lee index score and postoperative outcome is consistent. In addition, patients with MAEs also had more traditional factors, including age, male, and low BMI. A previous study showed that BMI showed a bimodal distribution with underweight and morbidly obese patients having increased mortality[19]. According to our data, lower BMI was associated with MAEs. Therefore, we chose a BMI < 19 kg/m2 as a factor in the multivariate analysis, and it was not an independent factor.

Our data demonstrated that at least moderate VHD, especially MR, was an independent predictor of perioperative MAEs. Patients undergoing noncardiac surgery with severe aortic stenosis (AS) had a higher 30-day mortality and a higher rate of postoperative myocardial infarction.[20] However, in this study, only 15 patients had significant AS/mitral stenosis (MS), and no patient in the MAE group had significant valvular stenosis. The small number of cases may be the reason why significant valvular stenosis did not have statistical significance. Additionally, clinically significant valvular regurgitation, i.e., MR and aortic regurgitation (AR) was shown to increase the likelihood of cardiovascular adverse events (such as myocardial infarction, stroke or heart failure) and in-hospital mortality during major noncardiac surgery.[11, 21, 22] This is consistent with our results, and we speculate that significant MR may mainly increase heart failure hospitalization. However, formal echocardiography is costly and time consuming. Significant MR could also be identified by other methods, for example, by physical examination or bed-side or point-of-care ultrasound techniques.

Our data also showed that the modified Lee index was an independent predictor of perioperative MAEs. Preoperative moderate-advanced CKD had been reported as an independent prognostic factor that increases the perioperative risks of morbidity (e.g., myocardial infarction, stroke or heart failure), mortality and cost in patients undergoing major noncardiac surgery.[17, 23, 24] We demonstrated that Cr >2 was the most significant clinical variable associated with MAEs, and this confirmed and extended previous findings. In addition, CAD and HF, which are included in the Lee index, were also significantly associated with MAEs in the univariate analysis. According to our data, we recorded diabetes mellitus but not insulin-dependent diabetes mellitus, which may be the reason why diabetes mellitus was not found to be associated with MAEs.

Based on the American College of Cardiology and the American Heart Association guidelines, routine preoperative evaluation of LV function is not recommended.[11] However, a number of studies have reported a significant positive correlation between impaired LV systolic function and perioperative complications.[5–9, 25] These data suggest that preoperative LV systolic function assessment can predict perioperative outcomes and long-term mortality in patients undergoing major noncardiac surgery, especially those undergoing high-risk noncardiac surgery. Currently, a growing number of surgeons and anesthesiologists routinely request preoperative echocardiography to assess cardiac function, especially LVEF. However, we must recognize that the role of the preoperative TTE in assessing the cardiac risk of patients undergoing major noncardiac surgery has not been thoroughly illustrated. In this study, we demonstrated that routinely evaluating the LV systolic function of patients undergoing scheduled abdominal surgery and orthopedic surgery was not correlated with perioperative MAEs. We found that only 63 patients (3%) had impaired LV systolic function (LVEF < 50%), and among patients who had MAEs after surgery, less than 10% had reduced LVEF. Furthermore, only 5% of the patients with reduced LVEF (3/63, Table 3) had MAEs after major noncardiac surgery. Our data demonstrated that routine perioperative echocardiography to assess LV systolic function did not provide more information to predict the cardiac risks of major noncardiac surgery. This important finding agreed with the American College of Cardiology and the American Heart Association guidelines, which state that preoperative reduced LVEF has a low sensitivity for the prediction of perioperative cardiac adverse events (major heart failure).[11]

This study had some important limitations. First, this was a retrospective cohort study, and all patients who underwent preoperative echocardiography were included, which may have led to some selection bias. Only a few echocardiography scans were arranged prior to surgery due to the development of a new murmur or new decompensated heart failure. Most of the patients underwent echocardiography due to hypertension and possible hypertensive cardiovascular disease (HCVD). In addition, we believe that some surgeries for patients with abnormal preoperative echocardiography findings, such as severe AS, were cancelled, which may have affected the results. In addition, we do not have some subjective data or functional status. Therefore, we could not evaluate the SNQIP score. Second, only patients who underwent intra-abdominal surgery or orthopedic surgery were included in this study. Patients who underwent open chest surgery or neurosurgery were not included because of the small number of patients. Third, few variables were included in the multivariable logistic analysis because there were few events during the study period. Finally, the study population consisted of patients recruited from only two medical centers and may not fully represent the population receiving noncardiac surgeries.

### Conclusions

Preoperative identification of significant MR on TTE is associated with an increased number of MAEs at 56 days compared with that predicted by the modified Lee index alone in patients undergoing intermediate- or high-risk noncardiac surgery.

## Supporting information

S1 Dataset Pre-operative echocardiography study raw data. (XLSX)
